# Characterization of Binder Interactions in Recycled Hot-Mix Asphalt Mixtures: Blending and Diffusion of Aged and Virgin Asphalt During Mixing and Stockpiling

**DOI:** 10.3390/ma19061214

**Published:** 2026-03-19

**Authors:** Yuquan Yao, Shiji Cao, Jiangang Yang, Jie Gao, Jiayun Xu, Jiayu Liu

**Affiliations:** 1 State Key Laboratory of Safety and Resilience of Civil Engineering in Mountain Area, East China Jiaotong University, Nanchang 330013, China; 3688@ecjtu.edu.cn (Y.Y.); gaojie@ecjtu.edu.cn (J.G.); 3738@ecjtu.edu.cn (J.X.); 2 Jiangxi Provincial Key Laboratory of Traffic Infrastructure Safety, East China Jiaotong University, Nanchang 330013, China; 3 School of Civil Engineering and Architecture, East China Jiaotong University, Nanchang 330013, China; 2024018085901130@ecjtu.edu.cn (S.C.); 2025018085901033@ecjtu.edu.cn (J.L.)

**Keywords:** RHAM, RAP, aged binder, blending degree, interfacial diffusion, production-process parameters

## Abstract

The performance of recycled hot-mix asphalt mixtures (RHAM) is strongly governed by the extent and uniformity of interactions between the aged binder in reclaimed asphalt pavement (RAP) and the virgin binder. However, in current engineering practice, it remains difficult to accurately evaluate the blending degree of aged and virgin asphalt during RHAM production, where the blending degree refers to the extent and uniformity of binder interaction during hot mixing. Moreover, influenced by various construction-related factors, the uniformity of interfacial diffusion between the two asphalt layers is also hard to control, which compromises the durability of RHAM. To address these issues, fluorescence microscopy was used to quantitatively characterize the blending behavior of aged and virgin asphalt, and Fourier transform infrared spectroscopy (FTIR) was employed to investigate the interfacial diffusion process and its evolution under time-temperature coupling conditions from plant production to field paving. The results indicate that, owing to the fluorescent characteristics of the Styrene-butadiene-styrene block copolymer (SBS) modifier in polymer-modified asphalt, the blending behavior during hot mixing can be quantitatively characterized by the fluorescent area and its areal proportion, providing a rapid solution for quantitative evaluation during RHAM production. Increasing the preheating temperature of RAP, extending mixing time, raising mixing temperature, and adopting Mixing Sequence I reduced the proportion of fluorescent area, suggesting improved blending between aged and virgin asphalt. After blending, the interfacial diffusion between aged and virgin asphalt occurs within the RHAM; the uniformity of this diffusion becomes more pronounced as the elapsed duration from production to paving increases. Nevertheless, excessively long duration may induce secondary aging of the blended binder. Accordingly, the duration is recommended to be controlled at approximately 90 min and should not exceed 180 min. By elucidating the blending and diffusion behaviors of aged and virgin asphalt, this study provides practical guidance for contractors in controlling production-process parameters for RHAM.

## 1. Introduction

As highway maintenance activities continue to expand and the scarcity of mineral aggregate resources becomes increasingly severe, the application of recycled hot-mix asphalt mixtures (RHAM) in road engineering has steadily grown. Efficient utilization of reclaimed asphalt pavement (RAP) materials can not only significantly reduce construction costs and decrease the consumption of natural aggregates and asphalt resources, but also help advance the vision of green and low-carbon development for transportation infrastructure [1,2,3]. Gkyrtis et al. [4] reported that RAP can be used as a supplement to virgin materials for highway maintenance and can also be applied in secondary road networks with lower traffic volumes. They suggested that RAP could be utilized in the upper layers of pavement structures; however, there remains uncertainty regarding the field performance of RHAM. The road performance of RHAM is mainly influenced by the combined effects of material composition and mixing processes [5,6,7]. Studies [8] have shown that the aged asphalt binder contained in RAP, obtained through milling of long-serving pavements, undergoes substantial deterioration in both mechanical and chemical properties. Consequently, achieving effective blending and synergistic interaction between the aged asphalt and virgin asphalt in RHAM has become a critical issue governing mixture performance.

During hot mixing, the aged asphalt mastic coating the surface of RAP particles gradually softens, flows, and migrates under heating and mechanical agitation, thereby interacting with the newly added virgin asphalt. However, substantial controversy remains regarding the extent of blending between aged and virgin asphalt and the spatial distribution of the blended asphalt on aggregate surfaces [9,10,11,12]. On the one hand, some researchers argue that aged and virgin asphalt can achieve relatively thorough blending at elevated temperatures. On the other hand, other studies suggest that the aged asphalt within RAP particles may participate only to a limited extent, resulting in a composite interfacial structure on aggregate surfaces with pronounced differences between aged- and virgin-asphalt-rich regions. Such interfacial heterogeneity can directly affect binder–aggregate adhesion as well as the mechanical performance and durability of RHAM. Overall, partial blending is now widely accepted as the prevailing outcome in the literature. In addition, to characterize the blending behavior of aged and virgin asphalt during hot mixing, Presti et al. [10] classified the aged asphalt on RAP surfaces into four types: liquid RA binder, softer RA binder, blackrock RA binder, and absorbed RA binder. Owing to the heterogeneous distribution of aged asphalt on RAP particle surfaces, under hot-mixing conditions, a portion of the aged asphalt mastic can detach and blend with virgin asphalt and rejuvenators, thereby contributing to the formation of RHAM [13]. Compared with virgin aggregates, the distinctive aggregate characteristics of RAP and the composition of its surface-aged asphalt make the phenomena occurring during heating and mixing with virgin aggregates, virgin asphalt, and rejuvenators highly complex. Based on a comprehensive literature review [14,15,16,17,18,19,20] from the perspective of RHAM, the factors reported to influence aged-virgin asphalt blending include: mixing temperature; the elapsed duration from production to paving; mixing time; RAP content; the shape characteristics of virgin aggregates; the type and dosage of additives such as rejuvenators; virgin asphalt properties; gradation type; water absorption and surface texture of virgin aggregates; aggregate source; and particle size distribution. Collectively, these factors can be grouped into two categories: mixing/process conditions and material composition. The former comprises mixing temperature, mixing time, RAP preheating temperature, mixing sequence, and the production-to-paving duration, whereas the latter includes RAP content, virgin-aggregate shape characteristics, the type and dosage of additives (e.g., rejuvenators), virgin asphalt properties, gradation type, water absorption and surface structure of virgin aggregates, aggregate source, and particle size distribution. These observations indicate that the blending behavior of aged and virgin asphalt in RHAM is governed by the coupled effects of multiple factors. Therefore, when designing RHAM, it is essential to comprehensively consider these coupled effects and to identify an optimal combination of material constituents and process parameters, so as to achieve recycled mixtures with balanced and superior overall performance.

Blending between aged and virgin asphalt occurs primarily during the mixing stage of recycled asphalt mixture production and is jointly governed by mixture composition and mixing/process conditions. After mixing, RHAM in practice typically undergoes a sequence of stages, including short-term storage in the hot bin at the asphalt plant, hauling from the plant to the field, on-site waiting prior to paving, and subsequent paving and compaction. These steps are commonly referred to as the post-production stage [21]. During this period, because of differences in molecular weight and composition between aged and virgin asphalt, interfacial diffusion occurs within the blended asphalt. In particular, throughout post-production, the mixture experiences continuous cooling while remaining at relatively elevated temperatures; under such conditions, the coupled time–temperature effect may substantially influence the extent of interfacial diffusion as well as the propensity for secondary aging. Diffusion-driven redistribution of aged and virgin fractions can alter asphalt rheological and adhesive properties, thereby affecting the durability of RHAM. Cui et al. [22] investigated aged-virgin asphalt interfacial diffusion using molecular dynamics simulations and reported that higher temperatures increase the interfacial diffusion coefficient and improve distribution uniformity; moreover, the degree of interfacial blending was positively correlated with crack resistance. Ge et al. [23] showed that greater asphalt aging makes blending between aged and unaged asphalt more difficult, whereas elevated temperatures promote mutual diffusion and blending; a more uniform interfacial blend was associated with higher interfacial tensile strength. Zhang et al. [24] demonstrated that the blending degree between aged and unaged asphalt significantly affects the high- and low-temperature performance of recycled materials as well as their resistance to moisture damage. Tao et al. [25] further found that more uniform blending and diffusion between aged and virgin asphalt leads to improved low-temperature cracking resistance of RHAM. Therefore, a systematic understanding of the blending characteristics of aged and virgin asphalt during the mixing stage, together with the interfacial diffusion behavior during the post-production stage, is of considerable engineering significance for optimizing construction practices and enhancing the long-term field performance of RHAM.

In practical applications, a timely and reliable assessment of the blending degree between aged and virgin asphalt is essential for adjusting and optimizing production parameters of RHAM. However, most existing evaluation approaches—whether macro-, meso-, or micro-scale—typically rely on extensive laboratory testing and the development of dedicated analytical models, which are time-consuming and therefore difficult to implement as a rapid feedback tool in routine plant production. In addition, the post-production stage is characterized by highly variable durations and continuously changing temperatures, yet the corresponding control window remains unclear when two competing requirements must be simultaneously satisfied: improving interfacial diffusion uniformity between aged and virgin asphalt while avoiding excessive secondary aging of the blended asphalt. Consequently, two practical gaps still remain.

(1) A rapid, quantitative, and production-oriented strategy for evaluating the blending between aged and virgin asphalt binders is still lacking.
(2) The evolution of interfacial diffusion jointly driven by time and temperature after production has not been clearly elucidated, making it difficult to provide actionable guidance for process control.

To bridge these gaps, this study proposes a rapid quantification approach for blending evaluation and systematically investigates the interfacial diffusion behavior and its evolution during post-production, aiming to identify a feasible control range that balances diffusion uniformity and aging risk.

## 2. Materials and Methods

### 2.1. Experimental Materials

The materials used in this study included reclaimed asphalt pavement (RAP), virgin aggregates, 70# base asphalt, and a styrene-butadiene-styrene block copolymer (SBS) modifier. The virgin aggregates were washed and sieved, then separated stepwise according to sieve opening size. The measured properties of each aggregate size fraction are summarized in Table 1. The basic properties of the 70# base asphalt are presented in Table 2.

**Figure 1:**
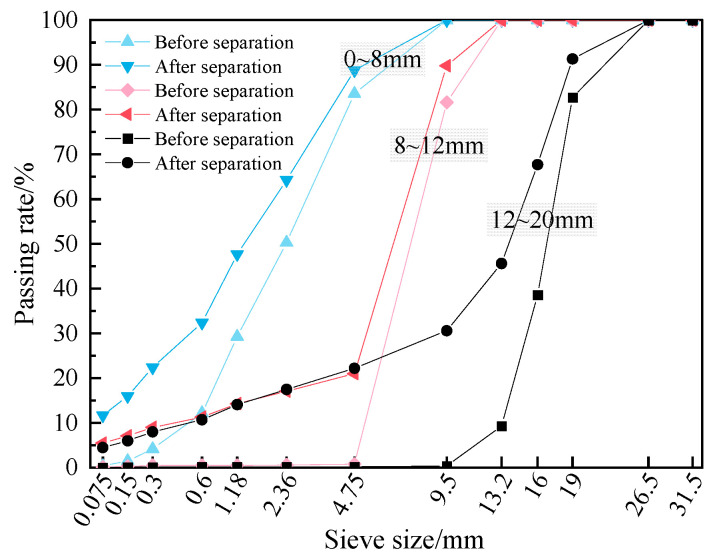
The gradation test results of RAP particles before and after extraction.

The RAP was obtained from a preventive maintenance project on an expressway in Jiangxi Province. To reduce particle agglomeration, the material was crushed and sieved, and then separated into three size fractions (0–8 mm, 8–12 mm, and 12–20 mm) for subsequent reuse. The particle gradation, aggregate gradation, and asphalt content of the RAP were determined using extraction and sieving methods. The gradation test results of RAP particles before and after extraction are shown in Figure 1. The measured asphalt binder contents of the 0–8 mm, 8–12 mm, and 12–20 mm RAP were 5.64%, 2.87%, and 2.70%, respectively. The aged asphalt was recovered from the RAP extract using rotary evaporation to separate the asphalt from the solvent. The basic properties of the recovered (aged) asphalt and the properties of the RAP aggregates were then measured; the results are reported in Table 3.

**Figure 2:**
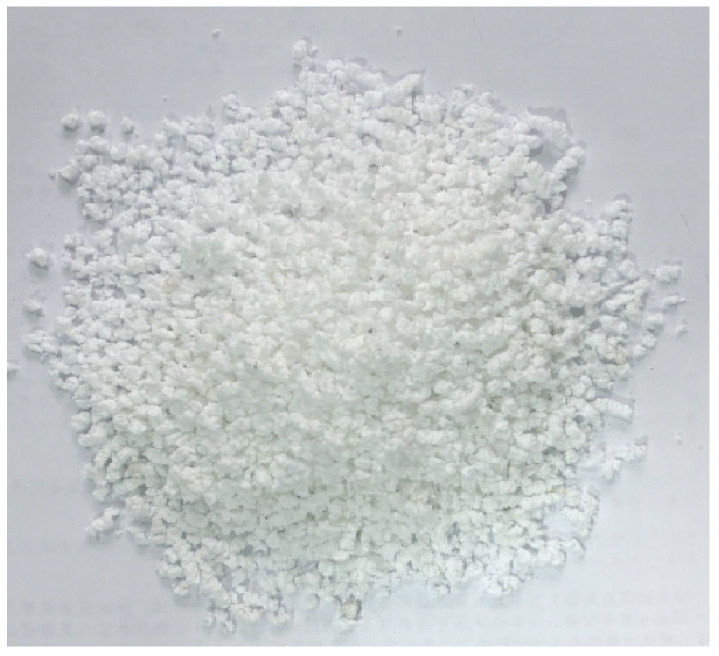
Appearance of the SBS modifier.

A linear SBS copolymer with a 30/70 block ratio and a relative molecular weight of 1.0 × 10^5^ was used. The SBS modifier was the T6302H grade manufactured by Xinjiang Dushanzi Petrochemical, Xinjiang Uygur Autonomous Region, China. Its macroscopic appearance is shown in Figure 2, and the detailed technical specifications are provided in Table 4.

### 2.2. Characterization of Virgin–Aged Asphalt Blending Based on Fluorescent Tracing

The aged asphalt coating the RAP particles was recovered by extraction followed by rotary evaporation. Fluorescence microscopy showed that the recovered aged asphalt exhibited no fluorescence. Therefore, to visualize how virgin and aged asphalt redistribute on aggregate surfaces during hot mixing, an SBS-modified asphalt was prepared using an SBS modifier and 70# base asphalt. To minimize the influence of rheological differences between the aged binder and the virgin binder, the aged asphalt in RAP was first recovered, and its kinematic viscosity was measured. Subsequently, SBS was incorporated into the 70# base asphalt so that the viscosity of the modified virgin binder was comparable to that of the recovered aged binder. Under this viscosity-equivalent condition, recycled asphalt mixtures were prepared and mixed to investigate the feasibility of using fluorescence microscopy to evaluate the blending behavior between aged and virgin binders during the hot-mixing process. This approach allows the fluorescent area and its areal proportion to primarily reflect the blending state of the two binders, thereby minimizing the influence caused by the difference in asphalt fluidity [26].

**Figure 3:**
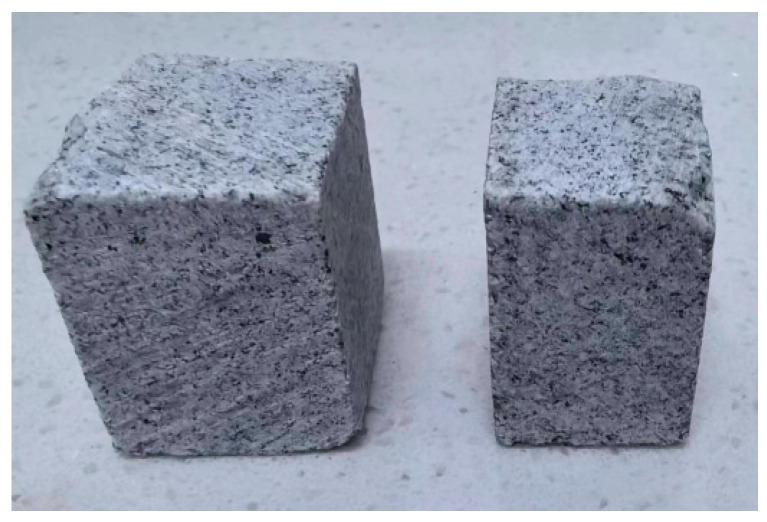
Cubic aggregate.

RAP-like particles were produced by mixing aggregates with the fluorescently traceable surrogate aged asphalt. These particles were then mixed with real RAP, virgin aggregates, and virgin asphalt to prepare RHAM. By tracking changes in the surface fluorescence of the surrogate asphalt RAP particles, the distribution of the blended asphalt on aggregate surfaces was evaluated. To clearly distinguish the surrogate asphalt RAP particles from the real RAP in the mixture, cubic aggregates (Figure 3) were used to fabricate the surrogate asphalt RAP particles.

**Figure 4:**
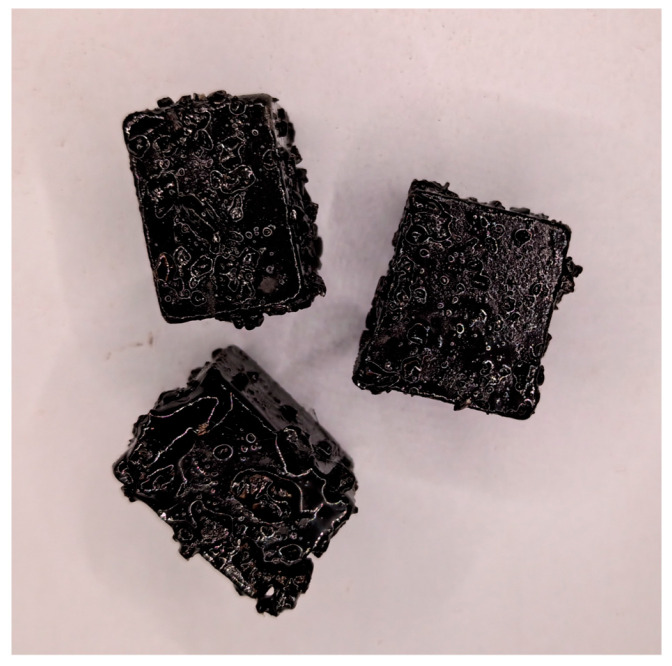
Cubic aggregate coated with fluorescent tracer asphalt.

Previous studies have shown that asphalt film thickness on aggregate surfaces influences the extent of blending between virgin and aged asphalt in RHAM [27]. In conventional hot-mix asphalt, the average asphalt film thickness is typically 5–15 μm [28]. Accordingly, a target film thickness of 10 μm was selected for the surrogate aged asphalt applied to the cube-shaped aggregates. Using the Hveem method [29], the required asphalt mass was determined from the aggregate specific surface area used in mix design, resulting in cubic aggregates coated with the surrogate aged asphalt (Figure 4).

The preparation of RHAM requires the addition of virgin aggregates and virgin asphalt. To avoid fluorescence interference that would occur if an SBS-modified asphalt were used as the virgin asphalt, 70# base asphalt was selected. This asphalt was used to enable a clear comparison of the post-mixing distribution of the blended asphalt on the surfaces of the cube-shaped aggregates.

**Figure 5:**
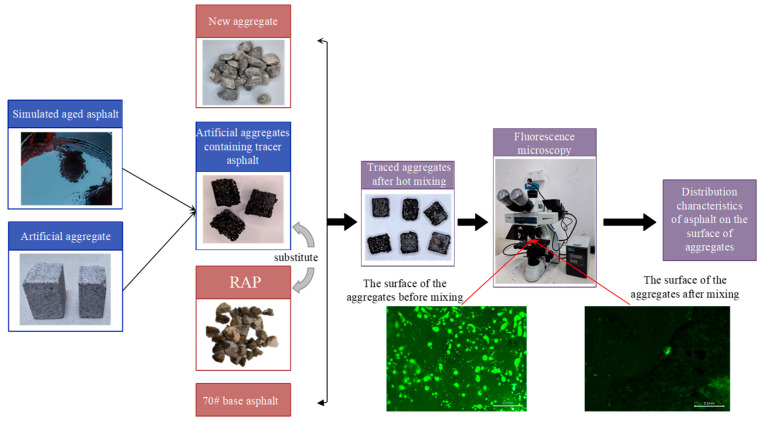
Testing program.

**Figure 6:**
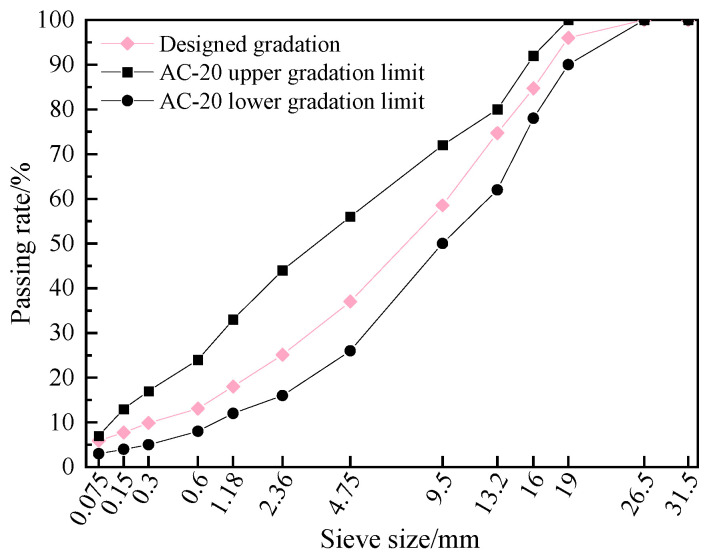
Designed gradation curve.

To characterize the distribution of the blended asphalt on aggregate surfaces after hot mixing, it is important to recognize that this behavior is jointly influenced by mixture composition and mixing conditions. Evaluating all interacting factors simultaneously would make it difficult to isolate individual effects and would require an impractically large experimental program. Therefore, a single-factor control variable method was adopted, focusing primarily on how mixing parameters affect the blending between virgin and aged asphalt. The experimental plan is provided in Table 5, and the workflow is shown in Figure 5. In all mixtures, the RAP content was fixed at 40%. The target gradation is shown in Figure 6, and the total asphalt content was 4.3%.

According to the experimental program, four cube aggregates coated with fluorescent tracer asphalt were added to each mixture. After mixing, these tracer-coated aggregates were retrieved from the RHAM, and their surfaces were examined using fluorescence microscopy. The distribution of the blended asphalt was evaluated based on the spatial characteristics of the fluorescence observed on the aggregate surfaces.

### 2.3. Interfacial Diffusion Characteristics of Virgin and Aged Asphalt Blending

#### 2.3.1. Time-Temperature Coupling Effects on Interfacial Diffusion

Previous studies have shown that blending between virgin and aged asphalt occurs during hot mixing and is accompanied by interfacial diffusion [30]. According to Fick’s law of diffusion [31], this interfacial process is governed by the coupled effects of time and temperature. Diffusion may continue throughout construction and service until the asphalt at the interface reaches a uniform state.

**Figure 7:**
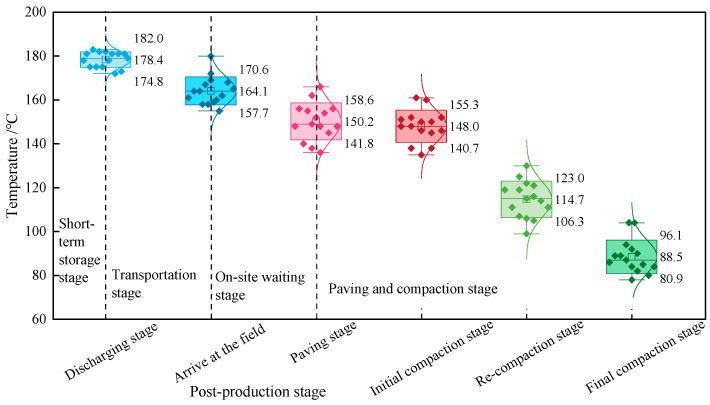
Temperature variation during the post-production stage of RHAM.

Because interfacial diffusion between virgin and aged asphalt is a relatively slow process, whereas the hot-mixing stage is brief, diffusion during mixing can be neglected. Based on previous work by the research team, the diffusion occurring after mixture production is defined as the post-production stage. This stage includes storage after plant production, transportation, on-site waiting, and paving and compaction. Field investigations were conducted to record temperature variations during the post-production stage, and the results are shown in Figure 7.

As shown in Figure 7, even for RHAM produced on the same day, different mixing batches exhibited different temperatures at key stages, including discharge, arrival on site, on-site waiting, and paving/compaction. The mean temperatures at discharge, arrival, on-site waiting, initial compaction, intermediate compaction, and final compaction were 178.4 °C, 164.1 °C, 150.2 °C, 148.0 °C, 114.7 °C, and 88.5 °C, respectively. Overall, temperature decreased progressively across the post-production stages. These observations indicate that interfacial diffusion within the blended asphalt can begin as early as the short-term storage period after mixing.

**Figure 8:**
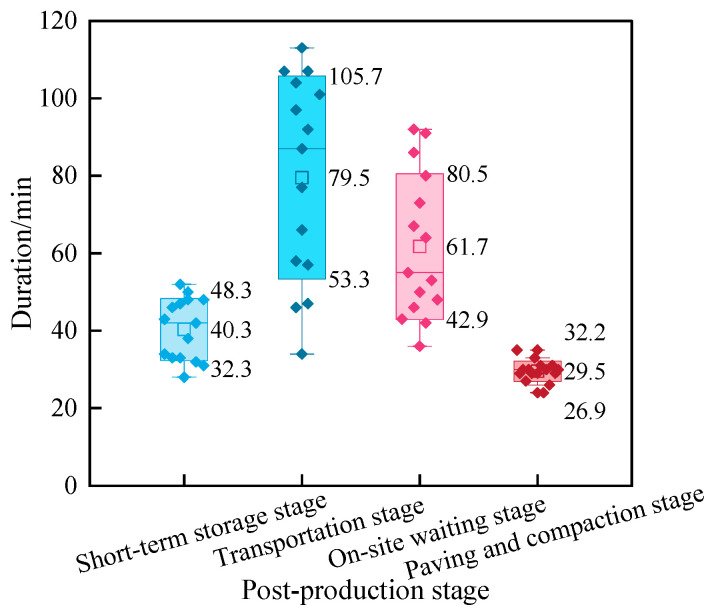
Duration variation during the post-production stage of RHAM.

The time spent at each construction stage is summarized in Figure 8. Short-term storage and paving/compaction were relatively brief, whereas transportation and on-site waiting lasted longer and showed greater variability. The mean duration for short-term storage, transportation, on-site waiting, and paving/compaction was 40.3 min, 79.5 min, 61.7 min, and 29.5 min, respectively. The corresponding maximum durations were 48.3 min, 105.7 min, 80.5 min, and 32.2 min, while the minimum durations were 32.3 min, 53.3 min, 42.9 min, and 26.9 min.

In summary, after mixing, RHAM is subjected to complex post-production conditions. The coupled effects of time and temperature during this period influence interfacial diffusion within the blended asphalt, which in turn affects mixture performance.

#### 2.3.2. Experimental Design for Interfacial Diffusion Characterization

Interfacial diffusion between virgin and aged asphalt depends on the degree of asphalt aging and on temperature and time conditions in the recycled mixture [25]. Given the fluctuations in temperature and duration during the post-production stage, this study focuses on diffusion occurring at relatively high temperatures during short-term storage, transportation, and on-site waiting. The mean temperatures of these three stages were used as representative temperature levels for evaluating temperature effects. The representative maximum, minimum, and average temperatures were 170.4 °C, 158.1 °C, and 164.2 °C, respectively. In addition, the observed duration ranges of the three stages were used as representative time levels to evaluate time effects on interfacial diffusion. The combined duration of these stages ranged from 128.5 to 234.5 min. Accordingly, a time-temperature coupling matrix (Table 6) was developed to investigate diffusion at the blended asphalt interface.

To investigate interfacial diffusion under time-temperature coupling conditions, this section excludes the effects of mixture composition and mixing procedures. Instead, it focuses solely on interfacial diffusion occurring after mixing has been completed. Based on the temperature and duration parameters listed in Table 6, ten experimental conditions were designed using an AC-20 recycled asphalt mixture. The corresponding gradation curve is shown in Figure 6. During mixture preparation, the RAP was preheated to 130 °C, the virgin aggregates to 190 °C, and the SBS-modified asphalt to 170 °C. The mixing temperature was 160 °C. Mixing Sequence I was adopted, and no rejuvenator was added. In Mixing Sequence I, each of the three mixing stages lasted 60 s, resulting in a total mixing time of 180 s. The designed asphalt content of the RHAM was 4.3%, and the total batch mass was 5.0 kg.

**Figure 9:**
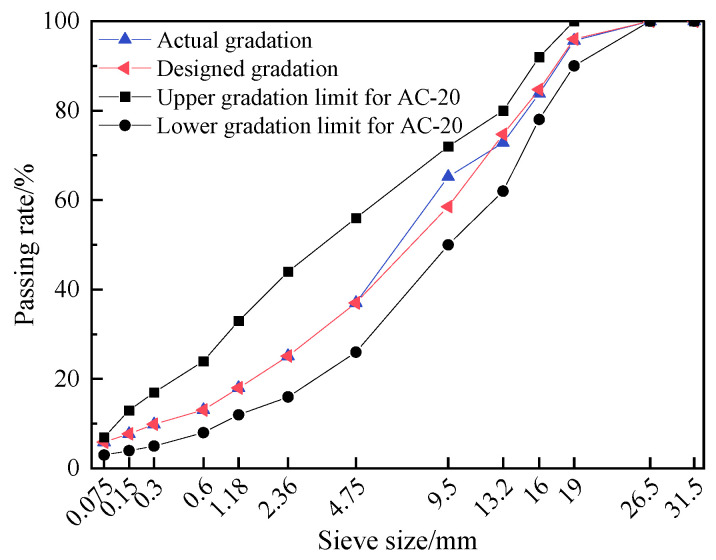
Gradation design results of the RHAM.

During mixture preparation, RAP particles needed to be accurately separated after being coated with the blended asphalt. Therefore, RAP with a particle size of 12–20 mm and virgin aggregates with a particle size of 0–9.5 mm were used, with each accounting for 50% of the aggregate mass. Due to the selected particle size ranges for RAP and virgin aggregates, the recycled mixture lacked part of the 9.5–16.0 mm fraction. As a result, slight differences existed between the actual and target gradations (Figure 9); however, the deviation was minimal. Given that the primary objective of this study was to examine interfacial diffusion under time-temperature coupling conditions, the minor gradation differences were considered to have negligible influence on the results.

**Figure 10:**
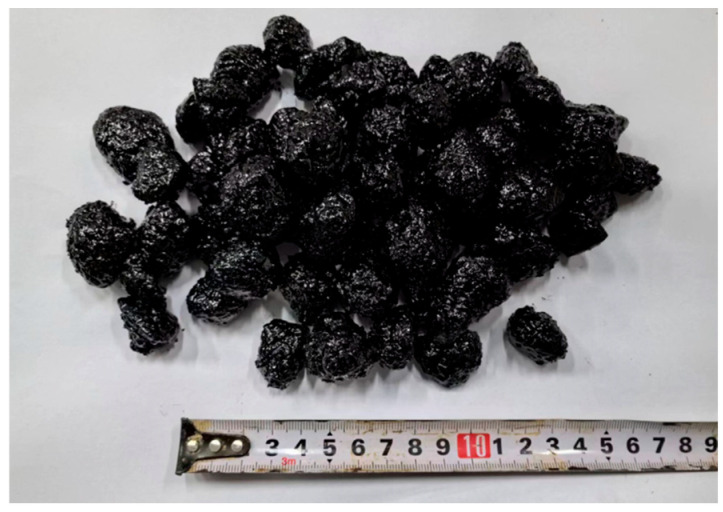
Separated RAP particles after screening.

After mixture preparation according to the experimental design, the blended mixture was sieved using a 13.2 mm square mesh sieve. RAP particles larger than 13.2 mm were collected after screening, as shown in Figure 10. A layered extraction method was then applied to separate the blended asphalt into three layers, enabling analysis of interfacial diffusion under different time-temperature coupling conditions.

Following the layered extraction procedure proposed by Bowers et al. [32], the blended asphalt was recovered in three stages. First, 800 g of RAP particles were placed in a mesh basket and immersed in 1.5 L of trichloroethylene (TCE) for 30 s to obtain the first-layer asphalt solution. The particles were then transferred to a second 1.5 L TCE bath and soaked for 3 min to collect the second-layer asphalt solution. Finally, the particles were immersed in a third 1.5 L TCE bath for 30 min to ensure complete removal of the remaining surface asphalt, yielding the third-layer asphalt solution. For each solution, mineral filler was removed by extraction, and the asphalt was separated from the solvent using rotary evaporation. This procedure produced three distinct asphalt layers for subsequent analysis.

Fourier transform infrared spectroscopy (FTIR) was conducted on the three recovered asphalt layers under each experimental condition. The carbonyl index was calculated using Equation (1) to quantify the effect of time-temperature coupling on interfacial diffusion between virgin and aged asphalt. Two parallel specimens were tested for each condition, and the average value was used to characterize diffusion at the blended asphalt interface.

(1) $$I_{C=O}=\frac{Area_{C=O}}{Area_{Reference}}$$

where *I_C_*_=*O*_ is the carbonyl index; *Area_C_*_=*O*_ is the area of the carbonyl functional group region. This area is determined by integrating the region enclosed between the upper (1722 cm^−1^) and lower (1672 cm^−1^) wavenumber limits of the carbonyl band. *Area_Reference_* is the area of the reference functional group region. This area is calculated by integrating the region enclosed between the upper (3026 cm^−1^) and lower (2766 cm^−1^) wavenumber limits of the reference band.

## 3. Results

### 3.1. Characterization of Blended Asphalt Distribution Based on Fluorescent Tracing

#### 3.1.1. Analysis of Blended Asphalt Distribution Characteristics

**Figure 11:**
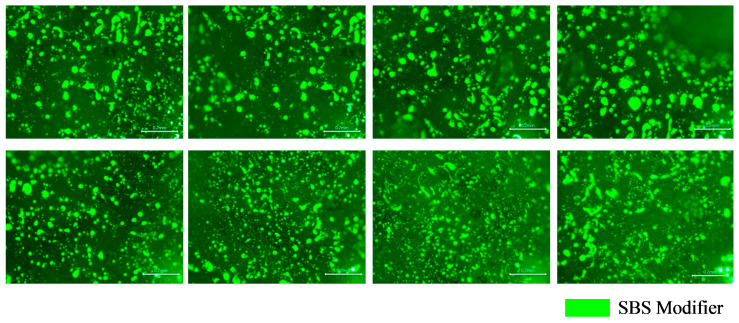
Fluorescence test results of tracer binder on the cubic aggregate surface before heating.

**Figure 12:**
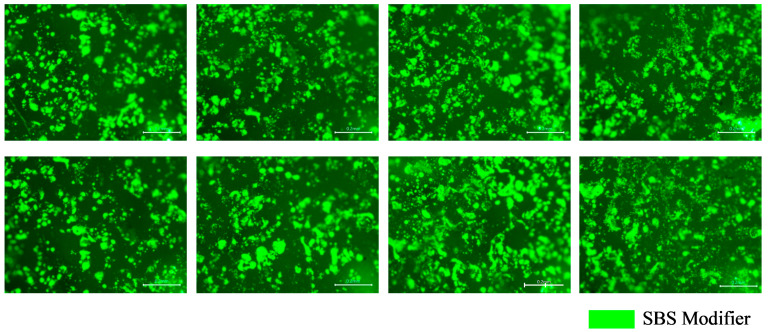
Fluorescence test results of tracer binder on the cubic aggregate surface after heating.

The fluorescence signal of the tracer binder coating the cube-shaped aggregates may change during heating, which could affect the interpretation of binder distribution after mixing. Therefore, fluorescence microscopy was used to capture the surface fluorescence characteristics of the cube aggregates before and after heating, as shown in Figure 11 and Figure 12.

As shown in Figure 11 and Figure 12, the cubic aggregates coated with tracer binder exhibited a clear fluorescence signal both before and after heating, attributable to the SBS modifier. The fluorescence images indicate that the SBS modifier was uniformly distributed within the tracer binder on the aggregate surface. After heating, the apparent fluorescent area slightly increased. Overall, no significant differences were observed in the fluorescence characteristics of the tracer binder before and after preheating, confirming that the fluorescent signal remained stable and distinct.

**Figure 13:**
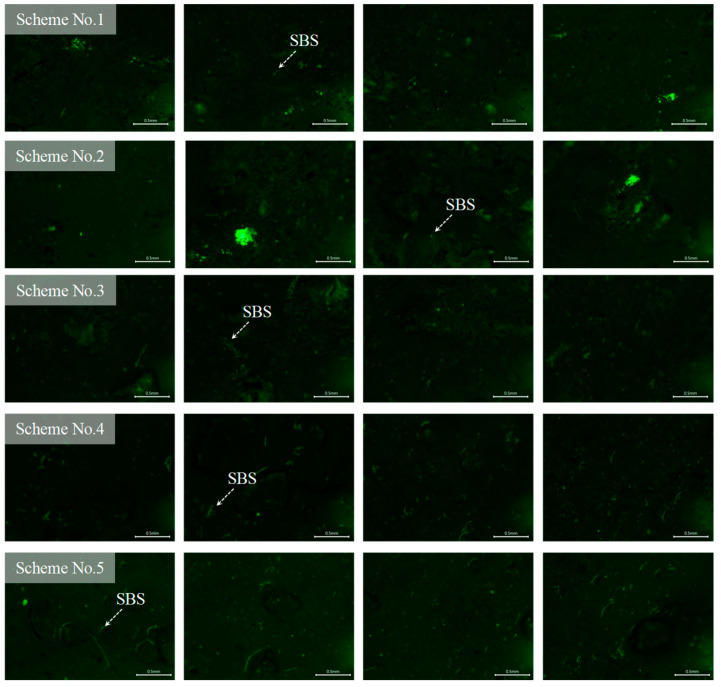

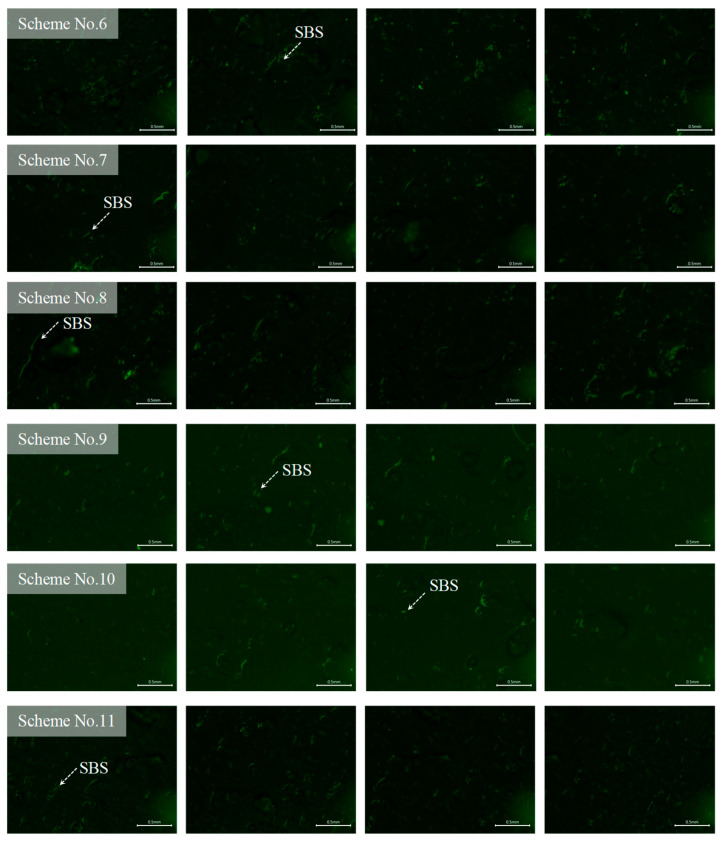
Fluorescence test results of blended binder on cubic aggregate surfaces under different experimental schemes.

**Figure 14:**
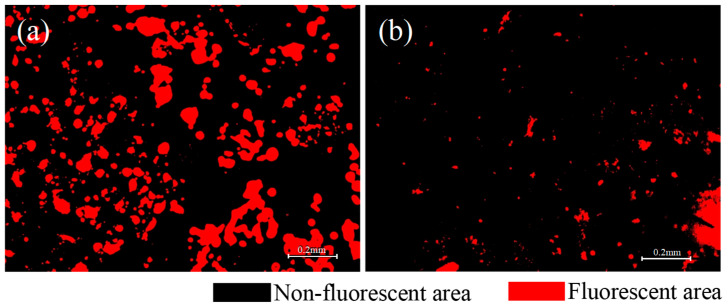
Fluorescent regions on the surface of the cubic aggregate. (**a**) After preheating the cubic aggregates, (**b**) the mixed cubic aggregates of Scheme 1.

The fluorescence distributions of the tracer binder on the cube-shaped aggregates under different experimental conditions are presented in Figure 13. The limestone cube-shaped aggregates, the aged binder in RAP, and the base asphalt exhibited no fluorescence. As shown in Figure 13, only partial fluorescence remained on the surface of the cube-shaped aggregates after hot mixing, while most areas appeared dark. This observation indicates that blending occurred between the aged binder in the RAP and the virgin binder during mixing. The interaction altered the surface binder properties of the tracer-coated aggregates, resulting in changes to the fluorescence signal. The fluorescent regions in Figure 12 and Figure 13 were identified using Image-Pro Plus 6.0 software to quantitatively compare the surface binder characteristics of the tracer-coated aggregates before and after mixing, as shown in Figure 14.

As shown in Figure 14, after mixing the RHAM, the size and area of fluorescent regions on the cubic aggregate surfaces decreased markedly. In the materials used in this study, neither the base asphalt nor the aged binder in RAP exhibited fluorescence. The fluorescence signal was provided solely by the SBS modifier in the tracer binder. Previous studies have demonstrated that the size and area of fluorescent regions can be used to represent the local concentration of SBS within the binder. Therefore, the observed reductions in fluorescent area and feature size before and after mixing indicate that the tracer binder on the cube aggregate surfaces blended with both the aged binder from the RAP and the virgin binder during hot mixing. The fluorescence patterns further reveal that the blended binder was highly non-uniformly distributed. Different locations on the aggregate surface contained different proportions of aged and virgin asphalt, leading to substantial spatial variation in binder properties.

Because interfacial interactions between aggregate and binder directly affect asphalt mixture performance, the observed spatial variability in blended-binder properties implies the presence of a complex adhesion interface on the aggregate surface. Such heterogeneity may play an important role in the deterioration of RHAM performance.

#### 3.1.2. Quantitative Evaluation of Blended Asphalt Distribution Characteristics

**Figure 15:**
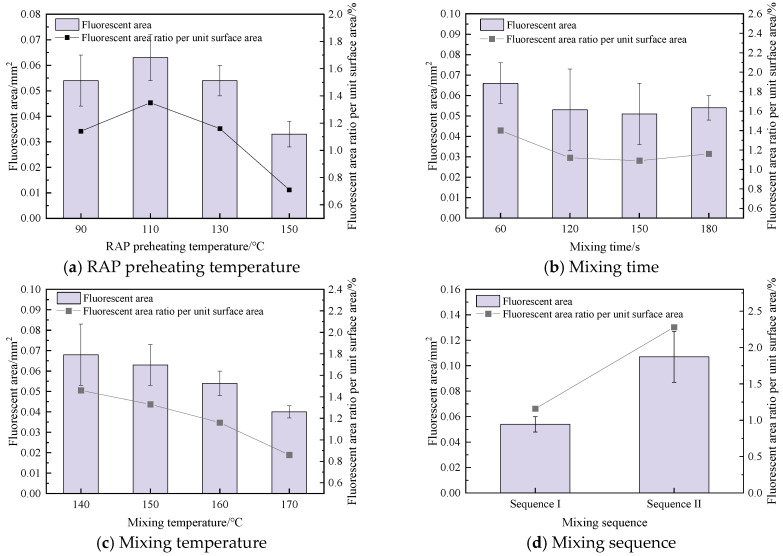
Quantitative analysis of the effects of mixing parameters on the fluorescence characteristics of the binder on cube-shaped aggregate surfaces.

Fluorescent regions were identified using Image-Pro Plus software. The area of each fluorescent region and its proportion relative to the total surface area were calculated to quantify the distribution of the SBS-containing tracer binder on the cubic aggregate surfaces after hot mixing. These metrics were used to evaluate the influence of mixture composition and mixing parameters on blended-binder distribution. The effects of different mixing parameters on the fluorescent area and its proportion of the total surface area are presented in Figure 15. A smaller fluorescent area and a lower fluorescent area ratio indicate more extensive blending between the SBS-containing tracer binder, the virgin binder, and the aged binder in RAP. In other words, reduced fluorescence corresponds to a higher degree of blending between virgin and aged asphalt. Conversely, larger fluorescent areas reflect a lower degree of blending in the RHAM.

As shown in Figure 15, the standard errors under different experimental schemes vary considerably. This is mainly attributed to the differences in the uniformity of the aged and virgin binders distributed on the RAP aggregate surface, which leads to relatively large deviations among the test results. It can therefore be inferred that the properties of the asphalt distributed at different locations on the RAP surface may vary, indicating that the adhesion interface on RAP particle surfaces exhibits a certain degree of uncertainty. Nevertheless, this variability remains within an acceptable range, suggesting that the measurements demonstrate good repeatability. In addition, both the fluorescent area and the proportion of fluorescent area per unit surface decreased with increasing RAP preheating temperature, longer mixing time, and higher mixing temperature. This trend indicates more extensive blending between the SBS-containing tracer binder on the aggregate surface and both the aged binder in RAP and the virgin binder. Compared with Mixing Sequence I, Mixing Sequence II resulted in larger fluorescent areas and higher fluorescent area ratios, with an increase of up to 97.04%. This suggests that a greater proportion of tracer binder remained on the aggregate surface under Sequence II, indicating a lower degree of blending between virgin and aged asphalt.

In summary, variations in mixing parameters significantly affect the fluorescent area observed on the cube-shaped aggregate surfaces. After preheating, the fluorescent area ratio of the SBS-modified tracer binder on the cubic aggregate surface was 20.31%. Compared with this baseline, the fluorescent area ratio decreased markedly after mixing, confirming that binder blending occurred during hot mixing and altered the properties of the tracer binder on the aggregate surface.

### 3.2. Analysis of Interfacial Diffusion Characteristics of Blended Asphalt

According to the experimental design, the calculated carbonyl indices of the blended binder under different time–temperature coupling conditions are presented in Table 7. As indicated in Table 7, after mixture production, variations in conditioning temperature and conditioning time influenced the extent of interfacial diffusion in the blended binder. For the blended asphalt film on RAP aggregate surfaces, the outermost layer (Layer 1) exhibited a lower carbonyl index than the innermost layer (Layer 3). Because the carbonyl index reflects the degree of binder aging, this result indicates that Layer 3 was more aged than Layer 1. In other words, the proportion of aged binder in the outermost blended-binder layer was lower than that in the innermost layer. Therefore, the blended binder became progressively more aged closer to the RAP aggregate surface. This observation is consistent with the layered binder structure on RAP particles. Layer 1 represents the outermost binder film and is most likely to flow during hot mixing, allowing frequent contact with the virgin binder and promoting blending; consequently, Layer 1 shows the lowest degree of aging. In contrast, Layer 3 is closest to the RAP aggregate. Although it becomes molten during mixing, it is less prone to flow and has limited contact with the virgin binder. As a result, Layer 3 is dominated by the aged binder originally coating the RAP surface and thus exhibits a higher aging level.

To quantitatively analyze the influence of the coupling of time and temperature on the interface diffusion between aged and virgin asphalt. As shown in Table 7, at 160 °C, the ranges of carbonyl index across the three blended-binder layers at conditioning times of 0, 30, 90, 180, and 240 min were 0.00482, 0.00415, 0.00112, 0.00276, and 0.00283, respectively. At 170 °C, the corresponding ranges at 0, 30, 90, 180, and 240 min were 0.00482, 0.00439, 0.00103, 0.00225, and 0.00239, respectively. Compared with 0 min, the most pronounced increase in carbonyl index occurred at 30 min. At 160 °C, the increase rate reached 38.9–226.2%, while at 170 °C it reached 46.9–244.9%.

Under different conditioning temperatures, the differences in carbonyl index among the three blended-binder layers gradually decreased with increasing conditioning time. This trend indicates more uniform interfacial diffusion. At both conditioning temperatures, the three layers exhibited similar time-dependent trends. The largest differences in carbonyl index occurred at 0 min, while the smallest differences were observed at 90 min. However, when conditioning time was further extended, the differences among the three layers increased again, suggesting the onset of additional aging in the blended binder.

To further analyze the significance level of the influence of time and temperature on the interface diffusion between aged and virgin asphalt, a one-way analysis of variance (ANOVA) was conducted to evaluate the significance of conditioning temperature and conditioning time on the carbonyl index. The *p*-values for temperature and time were 0.910 and 0.004, respectively. These results indicate that conditioning time had a statistically significant effect on interfacial diffusion, whereas conditioning temperature did not.

Considering the time-temperature coupling effects and the potential for secondary aging, when conditioning temperatures of 160 °C or 170 °C are used, it is recommended that the combined duration of short-term storage, transportation, and on-site waiting be controlled within 90 min and not exceed 180 min. Under these conditions, diffusion between virgin and aged asphalt at the interface is relatively uniform, and the durability of the RHAM can be ensured.

## 4. Discussion

**Figure 16:**
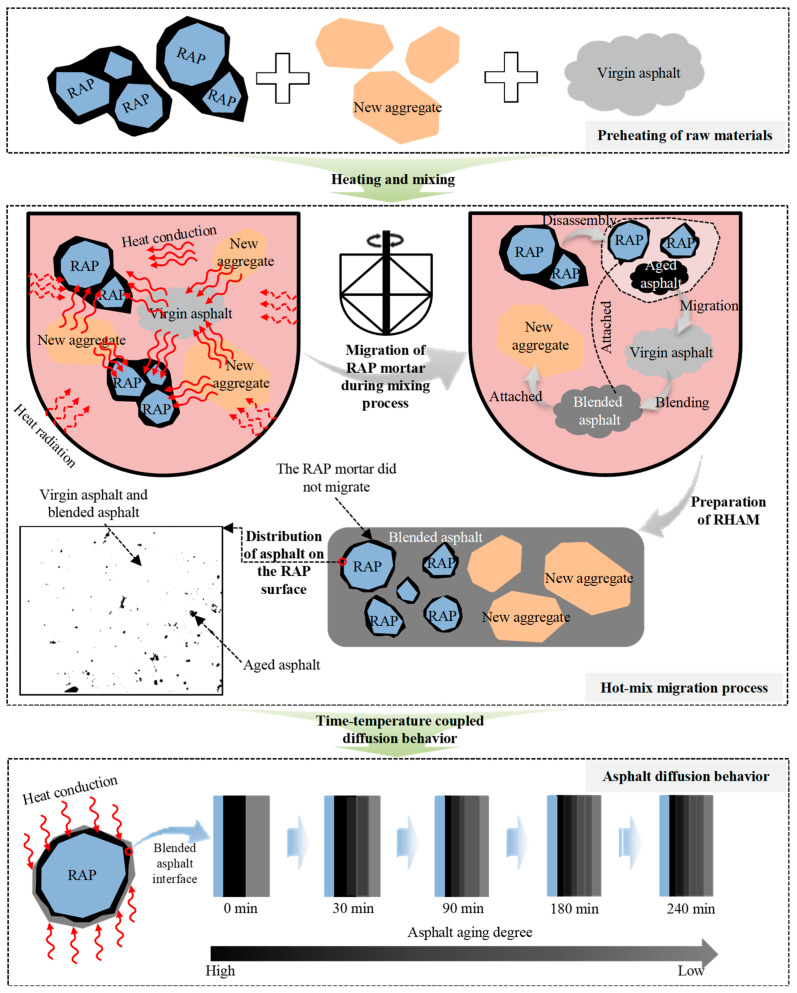
The influence mechanism of the new and old asphalt blending characteristics under the migration of RAP mortar.

During hot mixing, the aged asphalt mortar coating the RAP particles interacts with the virgin aggregates and virgin binder. As the temperature rises, the aged mortar gradually detaches from the RAP aggregate surface and begins to migrate. Simultaneously, it interacts with the virgin binder, forming blended binder films that coat both the virgin aggregates and the RAP particles. Based on the observed distribution of virgin and aged asphalt on RAP aggregate surfaces, together with blending test results, it was found that under different material compositions and mixing conditions, the aged asphalt mortar on RAP surfaces cannot completely blend with the virgin binder during hot mixing. The spatial distribution of blended binder under various mixture compositions and mixing parameters reveals pronounced non-uniformity. Different regions on the aggregate surface contain markedly different proportions of aged and virgin asphalt, resulting in a complex binder–aggregate adhesion interface. Therefore, from the perspective of aged mortar migration from RAP particles, the mechanism governing virgin–aged binder blending can be analyzed to clarify the dispersion behavior of RAP particles during hot mixing, as illustrated in Figure 16.

As shown in Figure 16, during mixing of RHAM, the temperature of RAP particles increases due to the elevated drum environment and heat exchange among materials. The aged asphalt mortar on the RAP surface gradually softens and becomes fluid. Under the mechanical action of the mixer, the softened mortar interacts with virgin aggregates and virgin binder, leading to mortar migration and simultaneous blending between aged and virgin asphalt. The migration of RAP mortar is governed by the combined effects of mixture composition and mixing parameters. Fundamentally, these factors determine the temperature achieved by RAP particles within the mixture, thereby controlling the extent of mortar migration during hot mixing.

During RAP mortar migration, blending between aged and virgin asphalt occurs simultaneously. The extent of blending depends on the migration rate of the RAP mortar; therefore, variations in mixture composition and mixing parameters directly influence the blending characteristics of virgin and aged asphalt RAP mortar. Migration also affects the spatial distribution of the blended binder on aggregate surfaces. Higher levels of mortar migration generally result in a more uniform binder distribution. The observed distribution patterns indicate that different regions of the aggregate surface contain varying proportions of virgin and aged asphalt, resulting in pronounced heterogeneity. This study defines this phenomenon as a complex binder–aggregate adhesion interface. Such interfacial complexity alters binder–aggregate interactions and may ultimately affect the performance of RHAM.

During hot mixing, blending between aged and virgin asphalt is driven by compositional differences between the aged mortar on RAP particles and the virgin binder. These differences lead to variations in intermolecular potential energy and van der Waals interactions, promoting the migration of lighter molecular fractions and initiating interfacial diffusion. Binder blending is accompanied by interfacial diffusion; however, diffusion is a continuous process that predominantly occurs during the post-production stage after mixing. This process is governed by the coupled effects of conditioning time and temperature. Conditioning temperature controls the mobility of lighter fractions between asphalt and can accelerate diffusion, whereas conditioning time determines the extent to which diffusion progresses toward equilibrium. It should be noted that although higher temperatures and longer conditioning times in the post-production stage may improve diffusion uniformity, excessive exposure can intensify secondary aging of the blended binder. Secondary aging of the blended binder can alter interfacial interactions between aggregate and binder, thereby reducing the long-term durability of RHAM.

In summary, this study has important practical implications for the production and application of RHAM. A rapid and quantitative method was developed to evaluate the blending behavior between aged and virgin asphalt during hot mixing, which provides a feasible basis for optimizing production parameters in engineering practice. Moreover, the results demonstrate that interfacial diffusion continues during the post-production stage and is strongly affected by time–temperature coupling conditions. To ensure the performance of RHAM, a certain period of storage and transportation after production is necessary. This period allows sufficient diffusion at the interface of the blended binders within the RHAM, thereby helping to ensure the stability of the mixture performance. Based on the aforementioned research findings, the recommended post-production duration of approximately 90 min, and not exceeding 180 min, may provide actionable guidance for plant production and field construction when using RAP in asphalt mixtures.

## 5. Conclusions

Based on a fluorescence-tracer methodology and a layered-extraction/FTIR approach, this study investigated the spatial heterogeneity of aged-virgin asphalt blending during hot mixing and the interfacial diffusion evolution of the blended binder during the post-production stage. The main conclusions are as follows:

(1) A rapid and quantitative characterization strategy for aged–virgin binder blending was established using the intrinsic fluorescent signature of the SBS modifier. After hot mixing, the fluorescent area and its areal proportion on aggregate surfaces decreased markedly and exhibited pronounced spatial non-uniformity, indicating that binder interaction is generally partial and that a complex, heterogeneous adhesive interface forms on aggregate surfaces.
(2) Mixing process parameters significantly affected the blending level. Increasing RAP preheating temperature, extending mixing duration, and raising mixing temperature consistently reduced the fluorescent-area proportion, demonstrating enhanced blending between aged and virgin asphalt. In contrast, mixing sequence II deteriorated blending, confirming that the process pathway plays a critical role in controlling interfacial heterogeneity.
(3) During the post-production stage, aged-virgin binder interfacial diffusion proceeded within the blended binder, as evidenced by carbonyl index gradients obtained from three-layer extraction. With increasing holding time, the interlayer differences in carbonyl index tended to decrease, suggesting improved diffusion uniformity; however, excessively long holding promoted secondary aging, which may impair binder performance.
(4) The coupled time-temperature analysis indicated that diffusion uniformity was governed primarily by holding time, whereas the effect of holding temperature was comparatively limited within the investigated range. Balancing diffusion uniformity and secondary-aging risk, the total high-temperature post-production duration is recommended to be controlled at approximately 90 min, and should not exceed 180 min, providing practical guidance for process control from production to paving in recycled asphalt mixture construction.
(5) It should be noted that the conclusions of this study are limited to the specific materials and experimental conditions investigated. Variations in asphalt binder type, modifier type, aggregate source, or RAP content may affect the blending and diffusion behavior between aged and virgin asphalt. Therefore, further studies considering a wider range of materials and mixture compositions are necessary to verify the general applicability of the proposed method.

## Figures and Tables

**Table 1:** Test results for the technical specifications of new aggregates.

Aggregate Size (mm)	Crush Value (%)	Apparent Relative Density	Flaky and Elongated Particles (%)	Adhesiveness
19–26.5	—	2.720	9.4	5 grade
16–19	—	2.714	8.6	5 grade
13.2–16	—	2.731	10.2	5 grade
9.5–13.2	13.4	2.695	13.5	—
4.75–9.5	—	2.738	16.3	—
2.36–4.75	—	2.726	—	—
1.18–2.36	—	2.711	—	—
0.6–1.18	—	2.717	—	—
0.3–0.6	—	2.721	—	—
0.15–0.3	—	2.709	—	—
0.075–0.15	—	2.733	—	—
Technical requirements	≤28	≥2.5	particle size > 9.5, ≤15	≥4 grade
			particle size < 9.5, ≤20	
Test Method	T 0316	T 0304	T 0312	T 0616

**Table 2:** Basic properties of 70# base asphalt.

Test Item	Result	Test Method
Penetration at 25 °C (0.1 mm)	60.8	T 0604
Softening point (°C)	50.7	T 0606
Ductility at 15 °C (cm)	>100	T 0605
Density at 25 °C (g/cm^3^)	1.025	T 0603
Solubility (%)	99.9	T 0607
Dynamic viscosity at 60 °C (Pa·s)	268.8	T 0625

**Table 3:** Basic properties of RAP particles.

Material	Test Item	Result	Test Method
RAP	Moisture content (%)	0.42	T0307 and T0334
	Sand equivalent (%)	78.9	
Asphalt in RAP	Penetration at 25 °C (0.1 mm)	31.2	T 0604
	Softening point (°C)	68.9	T 0606
	Ductility at 5 °C (cm)	Brittle fracture	T 0605
	Viscosity at 135 °C (Pa·s)	2.035	T 0620
Coarse aggregate in RAP	Crushing value (%)	13.2	T 0316
	Flakiness and elongation index (%)	14.3	T 0312
Fine aggregate in RAP	Angularity	30.8	T 0345

**Table 4:** Basic properties of SBS modifier.

Test Item	Specification Requirements	Result
Volatile matter (%)	≤1.0	0.12
Total styrene content (%)	28–32	30.6
Shore hardness (Shore A)	≥88	91

**Table 5:** Experimental program.

Scheme No.	RAP Preheating Temperature (°C)	Mixing Time (s)	Mixing Temperature (°C)	Mixing Sequence
1	90	180	160	Sequence I
2	110	180	160	
3	130	180	160	
4	150	180	160	
5	130	60	160	
6	130	120	160	
7	130	150	160	
8	130	180	140	
9	130	180	150	
10	130	180	170	
11	130	180	160	Sequence II

Note: In mixing sequence I, RAP was first pre-mixed with the rejuvenator; virgin aggregates and virgin asphalt were then added and mixed, followed by the addition of mineral filler for final mixing. In mixing sequence II, RAP, virgin aggregates, rejuvenator, and virgin asphalt were mixed initially, and mineral filler was added afterward for final mixing.

**Table 6:** Temperature and duration parameters for conditioning of RHAM.

Temperature Parameters (°C)		Duration Parameters (min)				
Parameter 1	Parameter 2	Parameter 1	Parameter 2	Parameter 3	Parameter 4	Parameter 5
160	170	0	30	90	180	240

**Table 7:** Calculated carbonyl index of blended binder after layered extraction.

Temperature (°C)	Duration (min)	Layer	Carbonyl Index		
			Group 1	Group 2	Average
160	0	Layer 1	0.00109	0.00163	0.00136
		Layer 2	0.00222	0.00317	0.00270
		Layer 3	0.00686	0.00550	0.00618
	30	Layer 1	0.00426	0.00462	0.00444
		Layer 2	0.00690	0.00649	0.00669
		Layer 3	0.00871	0.00845	0.00858
	90	Layer 1	0.00619	0.00575	0.00597
		Layer 2	0.00579	0.00707	0.00643
		Layer 3	0.00696	0.00721	0.00708
	180	Layer 1	0.00667	0.00558	0.00612
		Layer 2	0. 00731	0.00744	0.00738
		Layer 3	0.00877	0.00899	0.00888
	240	Layer 1	0.00640	0.00599	0.00620
		Layer 2	0.00784	0.00798	0.00791
		Layer 3	0.00910	0.00895	0.00902
170	0	Layer 1	0.00109	0.00163	0.00136
		Layer 2	0.00222	0.00317	0.00270
		Layer 3	0.00686	0.00550	0.00618
	30	Layer 1	0.00448	0.00491	0.00469
		Layer 2	0.00726	0.00690	0.00708
		Layer 3	0.00917	0.00899	0.00908
	90	Layer 1	0.00703	0.00661	0.00682
		Layer 2	0.00650	0.00768	0.00709
		Layer 3	0.00740	0.00829	0.00785
	180	Layer 1	0.00775	0.00627	0.00701
		Layer 2	0.00881	0.00792	0.00837
		Layer 3	0.00895	0.00956	0.00926
	240	Layer 1	0.00762	0.00689	0.00726
		Layer 2	0.00871	0.00849	0.00860
		Layer 3	0.00989	0.00942	0.00965

## Data Availability

The original contributions presented in this study are included in the article. Further inquiries can be directed to the corresponding author.

## Abbreviations

The following abbreviations are used in this manuscript:

RHAM	recycled hot-mix asphalt mixtures
RAP	reclaimed asphalt pavement
SBS	Styrene-butadiene-styrene block copolymer
FTIR	Fourier transform infrared spectroscopy
